# Plastid translocon recycling in dinoflagellates demonstrates the portability of complex plastids between hosts

**DOI:** 10.1016/j.cub.2024.10.034

**Published:** 2024-11-20

**Authors:** William H. Lewis, Giulia Paris, Girish Beedessee, Ludek Kořený, Victor Flores, Tom Dendooven, Benoit Gallet, Daniel P. Yee, Simon Lam, Johan Decelle, Ben F. Luisi, Ross F. Waller

**Affiliations:** 1 Department of Biochemistry, University of Cambridge, Tennis Court Road, Cambridge CB2 1QW, UK; 2 MRC Laboratory of Molecular Biology, Francis Crick Ave, Trumpington, Cambridge CB2 0QH, UK; 3 Cell and Plant Physiology Laboratory, University of Grenoble Alpes, CNRS, CEA, INRAE, and IRIG, 17 Avenue des Martyrs, Auvergne-Rhone-Alpes, Grenoble 38054, France

## Abstract

The plastids of photosynthetic organisms on land are predominantly “primary plastids,” derived from an ancient endosymbiosis of a cyanobacterium. Conversely, the plastids of marine photosynthetic organisms were mostly gained through subsequent endosymbioses of photosynthetic eukaryotes generating so-called “complex plastids.” The plastids of the major eukaryotic lineages—cryptophytes, haptophytes, ochrophytes, dinoflagellates, and apicomplexans—were posited to derive from a single secondary endosymbiosis of a red alga in the “chromalveloate” hypothesis. Subsequent phylogenetic resolution of eukaryotes has shown that separate events of plastid acquisition must have occurred to account for this distribution of plastids. However, the number of such events and the donor organisms for the new plastid endosymbioses are still not resolved. A perceived bottleneck of endosymbiotic plastid gain is the development of protein targeting from the hosts into the new plastids, and this supposition has often driven hypotheses toward minimizing the number of plastid-gain events to explain plastid distribution in eukaryotes. But how plastid-protein-targeting is established for new endosymbionts is often unclear, which makes it difficult to assess the likelihood of plastid transfers between lineages. Here, we show that Kareniaceae dinoflagellates, which possess complex plastids known to be derived from haptophytes, acquired all the necessary protein import machinery from these haptophytes. Furthermore, cryo-electron tomography revealed that no additional membranes were added to the Kareniaceae complex plastid during serial endosymbiosis, suggesting that the haptophyte-derived import processes were sufficient. Our analyses suggest that complex red plastids are pre-adapted for horizontal transmission, potentially explaining their widespread distribution in algal diversity.

## Introduction

The gain and spread of photosynthesis in eukaryotes have demonstrated just how profoundly the process of endosymbiosis has shaped the evolution and diversity of complex cellular life on Earth.^1,2^ Although oxygenic photosynthesis first evolved in cyanobacteria, the endosymbiotic gain of photosynthetic organelles within eukaryotes—chloroplasts and other colored plastids—now means that most of the planet’s photosynthesis occurs in eukaryotic organisms. Primary plastids resulting from a single endo-symbiotic uptake from cyanobacteria are conspicuous in plants in the terrestrial environment, as well as in green and red algae in aquatic habitats. However, much more of the photosynthetic capacity of the marine environment is driven by organisms that have gained photosynthesis by further endosymbiotic events where an existing plastid-containing eukaryote was itself captured as a source of a new endosymbiont forming a so-called “complex plastid.” Such events can be secondary endosymbioses, where the new endosymbiont contained a primary plastid, or can be further serial endosymbiotic events creating tertiary and even higher-order endosymbioses. Complex plastids are responsible for photosynthesis in multiple major eukaryotic phyla: cryptophytes, haptophytes, ochrophytes (e.g., kelps and diatoms), dinoflagellates, euglenids, and chlorarachniophytes.^2^ Furthermore, some important phyla, such as the parasitic apicomplexans that cause diseases such as malaria, have lost photosynthesis but retain a complex plastid that continues to contribute to its core cellular metabolism.^3,4^ How many separate and/or serial endosymbiotic events that have occurred to create this diversity and the sources of complex plastids in each lineage have proven confounding problems to resolve.

Some earlier attempts to resolve the patterns of plastid inheritance across eukaryotes, that subsequently gained considerable traction in the field, favored minimizing the required number of endosymbiotic events. The “chromalveloate” and “cabozoan” hypotheses posited single secondary uptakes of either a red or green algae, respectively, giving rise to the multiple lineages with plastids that can be linked to these sources: red-algal-derived plastids in cryptophytes, haptophytes, ochrophytes, dinoflagellates, and apicomplexans and green-algal-derived plastids in euglenids and chlorarachniophytes.^5^ This proposed minimal number of endosymbiotic events explicitly sought to reduce the number of times that plastid protein-targeting processes for endosymbiont maintenance and coordination with the host would need to be independently gained, with the assumption that such a process would be complex and unlikely to evolve often. However, as phylogenomic studies of the eukaryotic tree of life have gained power and resolution, neither of the chromalveloate or cabozoan hypotheses could be sustained because neither of their constituent organisms have resolved as monophyletic lineages.^6,7^ Many alternative schemes of multiple secondary and further higher-order endosymbiotic transfers of plastids have sought to resolve the phylogenetic signals and incongruities of the organisms and their complex plastids.^6,8–12^ However, further questions that have remained unanswered and unaddressed are as follows: what processes are required to establish protein targeting to a new plastid endosymbiont, how difficult or likely are such processes to occur repeatedly, and how does this likelihood constrain or relax the number of possible independent transfers of plastids that we are prepared to consider in deciphering the evolutionary history of plastid-bearing eukaryotes?

Dinoflagellate algae, a major eukaryotic phylum and member of the former chromalveloate assemblage, offer an excellent opportunity to address the question of how protein targeting is established in new complex plastids. The ancestral plastid of dinofflagellates that occurs in most of its photosynthetic members contains a distinctive secondary pigment, the carotenoid peridinin.^13,14^ Many dinoflagellate groups, however, have more recently gained new plastids that replace the photosynthetic capacity of the “peridinin plastid.”^14^ The Kareniaceae are one such group with a proclivity for new plastid endosymbioses. Different taxa of Kareniaceae dinoflagellates are known to have different types of plastids: some have only the ancestral peridinin plastid (e.g., *Gertia stigmatica*),^15^ some continually acquire temporary kleptoplasts from haptophyte prey (e.g., an undescribed taxon, the Ross Sea Dinoflagellate [RSD]),^16,17^ and others have stable and fully integrated haptophyte-derived plastids (e.g., species of *Karlodinium, Karenia*, and *Takayama*).^18–20^ These different plastid states seemingly represent a continuum of replacing a photosynthetic peridinin plastid with a new haptophyte-derived plastid to fulfill the role of photosynthesis. The new stable plastids in *Karlodinium, Karenia*, and *Takayama* lack any vestige of haptophyte cytosolic organelles or nucleus^21^ and therefore must rely on protein import from their new host dinoflagellate for their biogenesis and function. These Kareniaceae taxa, therefore, offer an opportunity to determine how the gain of a system for protein import into new complex plastids has been achieved and to assess how complex and difficult such a process might or might not be such that it could hinder or facilitate plastid lateral transmission through eukaryotes.

## Results and Discussion

### Haptophyte translocons for all plastid membranes are maintained in Kareniaceae with stable haptophyte-derived plastids

**Figure 1:**
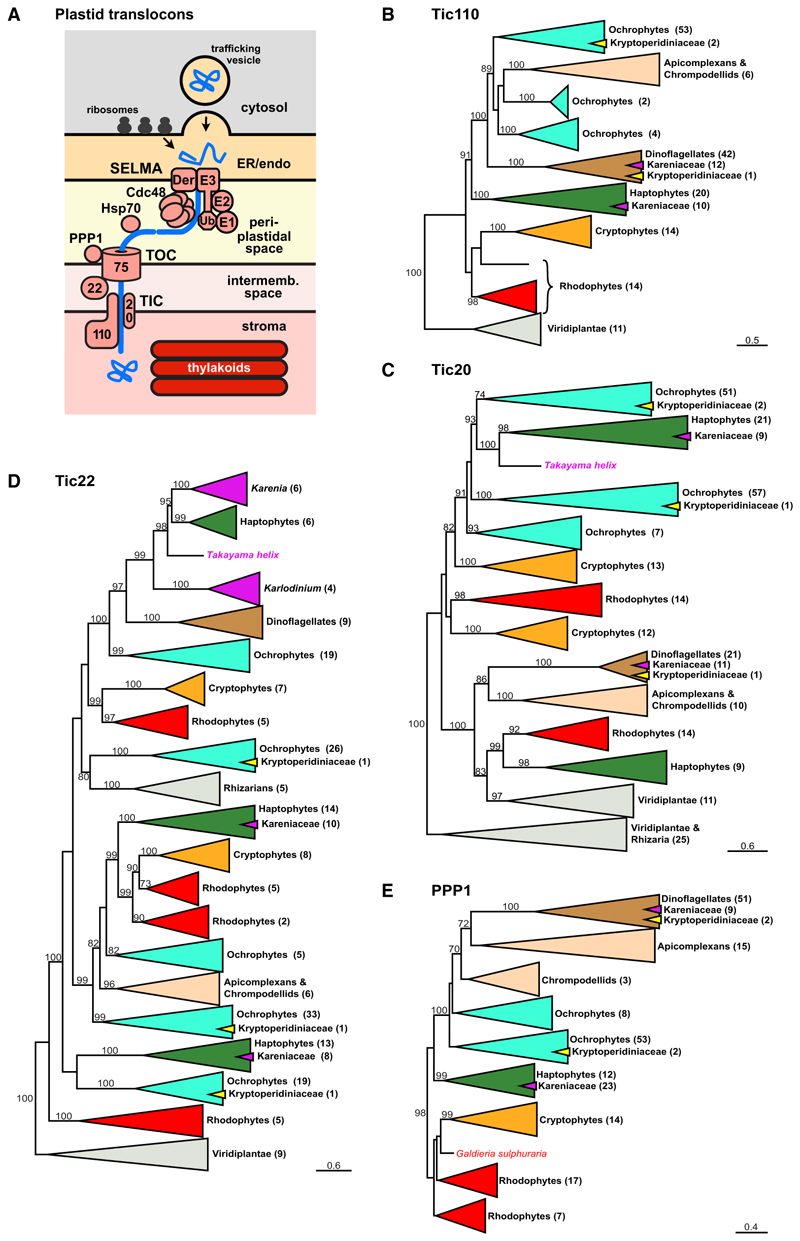
Inner membrane translocons are derived from haptophytes in Kareniaceae (A) Schematic of four-membrane-bound plastids and the protein translocation machinery conserved in haptophytes, ochrophytes, cryptophytes, and api-complexans. Protein delivery to the ER or endomembrane system (endo) can occur either via vesicular transport or, in some taxa, cotranslational import via ribosomes docked on the outer membrane. The symbiont-specific ERAD-like machinery (SELMA) transfers proteins into the periplastidal space as a relic of the red algal cytoplasm, and TOC and TIC derived from the primary plastid transfer proteins into the plastid stroma. (B–E) Protein maximum likelihood phylogenies of Tic110, Tic20, Tic22, and PPP1, respectively. Sequences found in the Kareniaceae monophyletic clades are indicated with magenta triangles or text. Dinotom sequences of the Kryptoperidiniaceae are shown with yellow triangles. Bootstrap support values >70 are shown and the number of sequences per clade is given in parentheses. Scale bars indicate estimates of amino acid substitutions per site. Full phylogenies are given in Data S1. See also Table S1.

The acquisition of new plastids in dinoflagellates of the Kareniaceae is sufficiently recent that the source organism(s) are clearly identified as haptophytes, and genes acquired with these haptophyte endosymbionts are readily discernible phylogenetically.^20^ Furthermore, the machinery for protein-targeting to haptophyte plastids is reasonably well studied and understood. Therefore, to investigate how Kareniaceae new plastids might subsequently perform this task, we asked how much of the haptophyte plastid-protein import machinery was inherited and redeployed in Kareniaceae dinoflagellates that might have facilitated the integration of these plastid endosymbionts in a new host. Plastids in haptophytes are surrounded by four membranes that use a system of four different translocons for the import of nucleus-encoded proteins for plastid function (Figure 1A).^22^ To test for the presence of haptophyte translocon components in Kareniaceae, we generated long- and short-read RNA sequencing (RNA-seq) data for *Karlodinium veneficum* and searched this and available transcriptomic data for two further *Karlodinium* spp. (*K. micrum* and *K. armiger*), three *Karenia* spp. (*K. brevis, K. mikimotoi*, and *K. papilionacea*), and *Takayama helix*. Phylogenies were constructed to test for the origin of these proteins.

The inner-most pair of membranes of most plastids are thought to be derived from the primary plastid endosymbiont. These membranes use conserved translocons of the inner and outer chloroplast membranes (TICs and TOCs, respectively) for protein import (Figure 1A).^22^ Tic110 and Tic20 are widely conserved inner membrane translocon components, and homologs were found throughout the Kareniaceae, which grouped specifically with haptophyte orthologs (Figures 1B and 1C, indicated as magenta within green triangles). A major pore-forming translocon element of the TOC complex is Toc75, but as a beta-barrel protein, it is often not well conserved or easily recognizable by primary sequence.^23^ We were not able to find homologs of Toc75 in the Kareniaceae nor any dinoflagellates. However, Tic22 is an intermembrane space protein that acts as a chaperone exchanging proteins from the TOC to TIC.^24^ Multiple paralogs of Tic22 are found throughout plastid types, and all Kareniaceae paralogs were found exclusively within or sister to the haptophyte paralog clades (Figure 1D). PPP1 is another—relatively uncharacterized—protein of the plastid import pathway, which in the apicomplexan *Toxoplasma* was verified as required for import of plastid proteins.^25^ In diatoms and apicomplexans, this protein is observed within the “periplastidal space,” which is the relic compartment of the red algal cytosol between the second and third membrane of complex plastids (Figure 1A).^25^ PPP1 is also present in red algae but not green algae or plants. Red algae contain two-membrane-bound primary plastids, and given that they lack the extra membranes of complex plastids, this implicates PPP1 with transport across the second membrane (counting from the inside) and as a component of the TOC. PPP1 homologs were found throughout Kareniaceae taxa, and these again grouped with haptophyte orthologs (Figure 1E). Collectively, these data indicate that elements of the haptophyte TIC and TOC complexes have been retained in the Kareniaceae.

**Figure 2:**
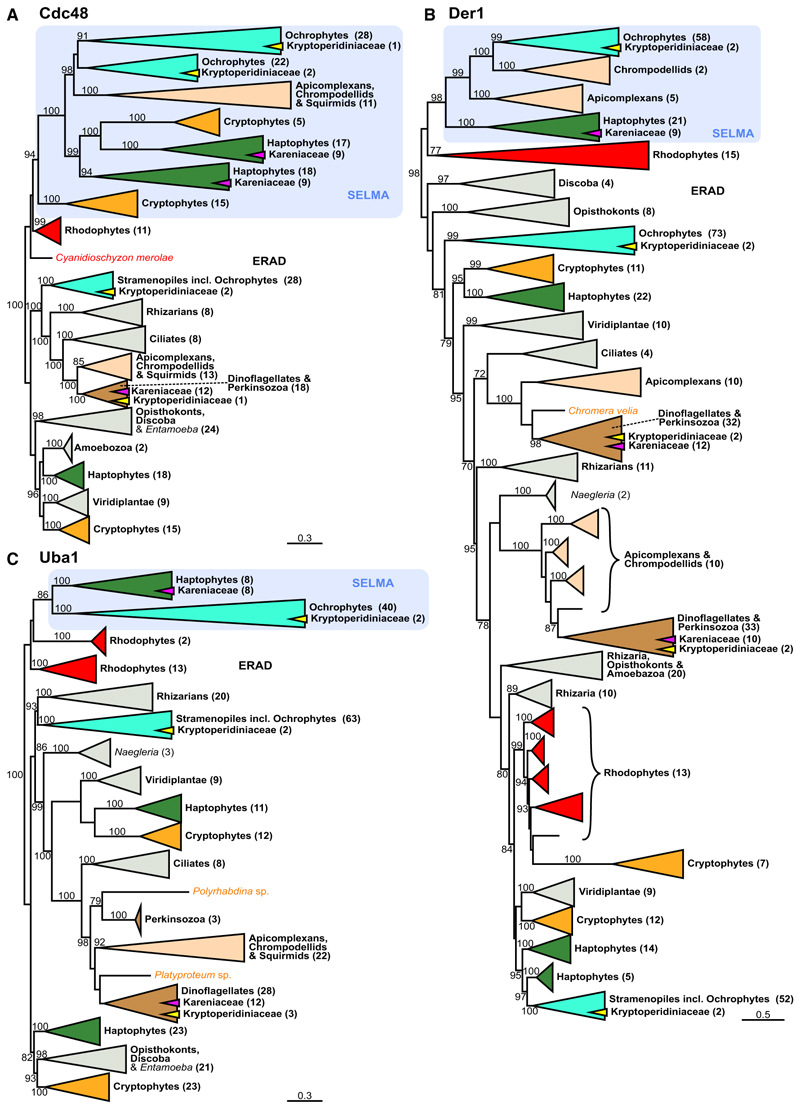
SELMA components Cdc48, Der1, and Uba1 are derived from haptophytes in Kareniaceae Protein maximum likelihood phylogenies of (A) Cdc48, (B) Der1, and (C) Uba1 shown as for Figure 1. Full phylogenies are given in Data S1. See also Table S1.

**Figure 3:**
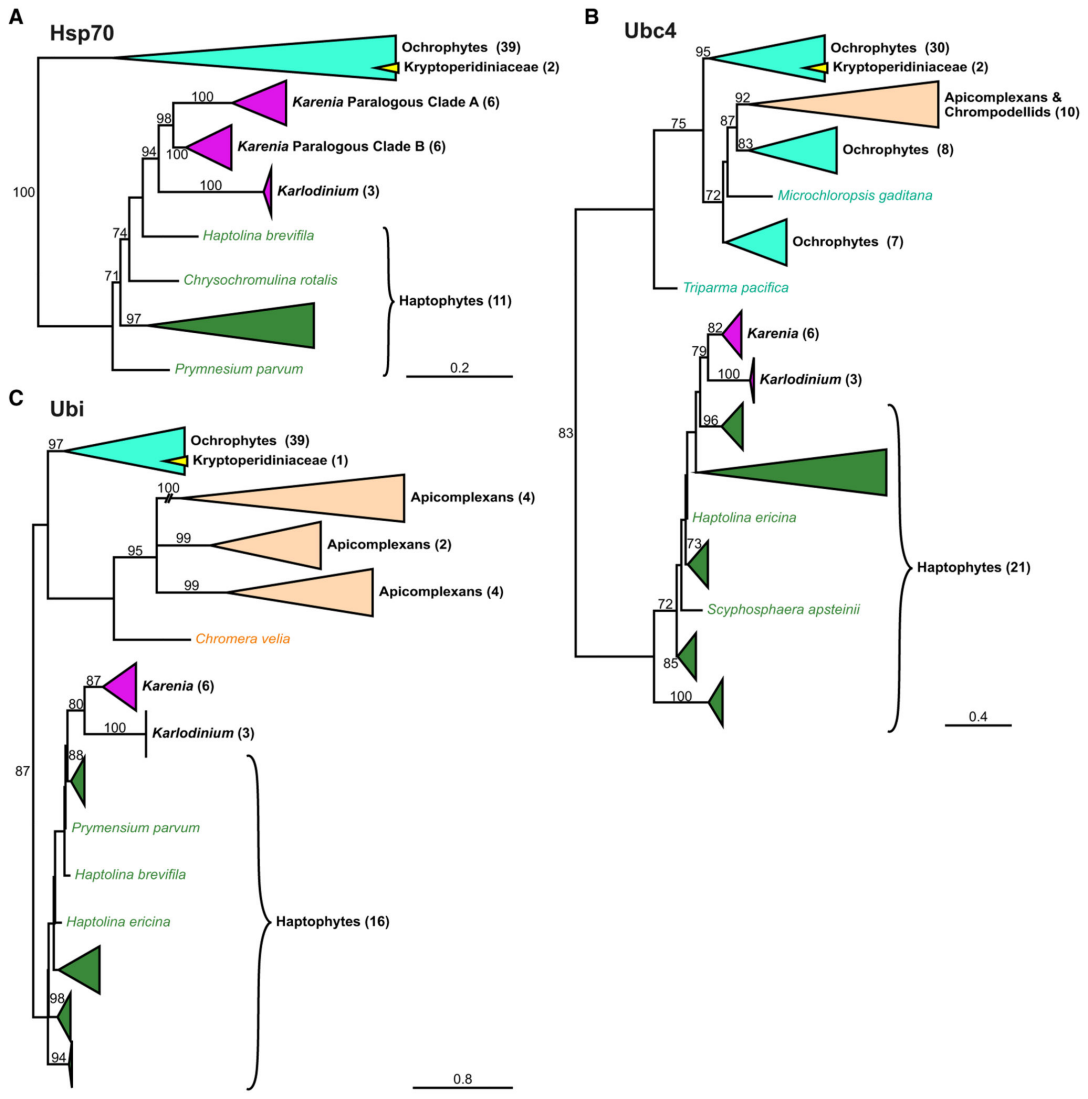
SELMA components Ubc4, Ubi, and Hsp70 are derived from haptophytes in Kareniaceae Protein maximum likelihood phylogenies of SELMA paralogs of (A) the chaperone Hsp70, (B) the ubiquitin conjugating enzyme Ubc4, and (C) ubiquitin (Ubi) are shown as in Figure 1. Full phylogenies are given in Data S1. See also Table S1.

The third plastid membrane (counting from the inside) of haptophytes, ochrophytes, cryptophytes, and apicomplexans uses a protein translocon system derived from the endoplasmic reticulum-associated degradation (ERAD) machinery that typically exports misfolded proteins from the ER to the cytoplasm for proteasomal degradation.^26–28^ In an ancestral secondary endosymbiosis of a red alga, most likely in a cryptophyte, the red alga’s ERAD was redeployed from the ER to its relict plasma membrane and repurposed to bring plastid proteins from the host inward into its cytoplasm (Figure 1A).^29,30^ Rather than be degraded, these proteins are then available to the plastid’s TOC and TIC for import into the plastid lumen. This symbiont-specific ERAD-like machinery (SELMA) consists of most of the ERAD elements: the derlin component of the membrane translocon, Der1; the ubiquitin-dependent AAA-ATPase Cdc48 that actively translocates the protein cargo; E1, E2, and E3 of the ubiquitination pathway; and chaperones to receive the disordered protein cargo.^26^ The development of this SELMA system to overcome the third membrane was a seminal event in the genesis of the red-algal-derived secondary plastid, enabling essential genes to relocate from the red algal nucleus to that of its new host. Given that ERAD is an essential element of the ER, eukaryotes with SELMA contain these proteins as a second paralogous machinery in their third plastid membrane. The molecular phylogenies of Cdc48, Der1, and Uba1 (the E1 ubiquitin activating enzyme) resolve the ERAD proteins separately from SELMA paralogs. The SELMA proteins all group within or sister to the red algal ERAD proteins, consistent with their origin from a red-algal-derived secondary plastid (Figure 2). Karenia-ceae dinoflagellates contain ERAD proteins that group within the rest of dinoflagellates, consistent with the expected vertical inheritance of this essential ER process. Additionally, SELMA paralogs from Kareniaceae dinoflagellates are found, and these group specifically within the haptophyte SELMA clades. In haptophytes, Cdc48 was duplicated, and the Kareniaceae dinoflagellates have inherited both Cdc48 SELMA paralogs (Figure 2A). Other ERAD/SELMA components include Ubc4 (the E2 ubiquitin conjugating enzyme), Ubi (the ubiquitin protein itself), and the chaperone Hsp70. These proteins all occur as multiple paralogs in most eukaryotes, and the phylogenetic histories of these paralogs are complex. However, all SELMA versions of these proteins in haptophytes have orthologs in *Karlodinium* and *Karenia* that group specifically within these haptophyte clades (Figure 3). Therefore, in addition to the TOC/TIC, the haptophyte SELMA protein import machinery for a third plastid membrane has also been retained in the Kareniaceae.

**Figure 4:**
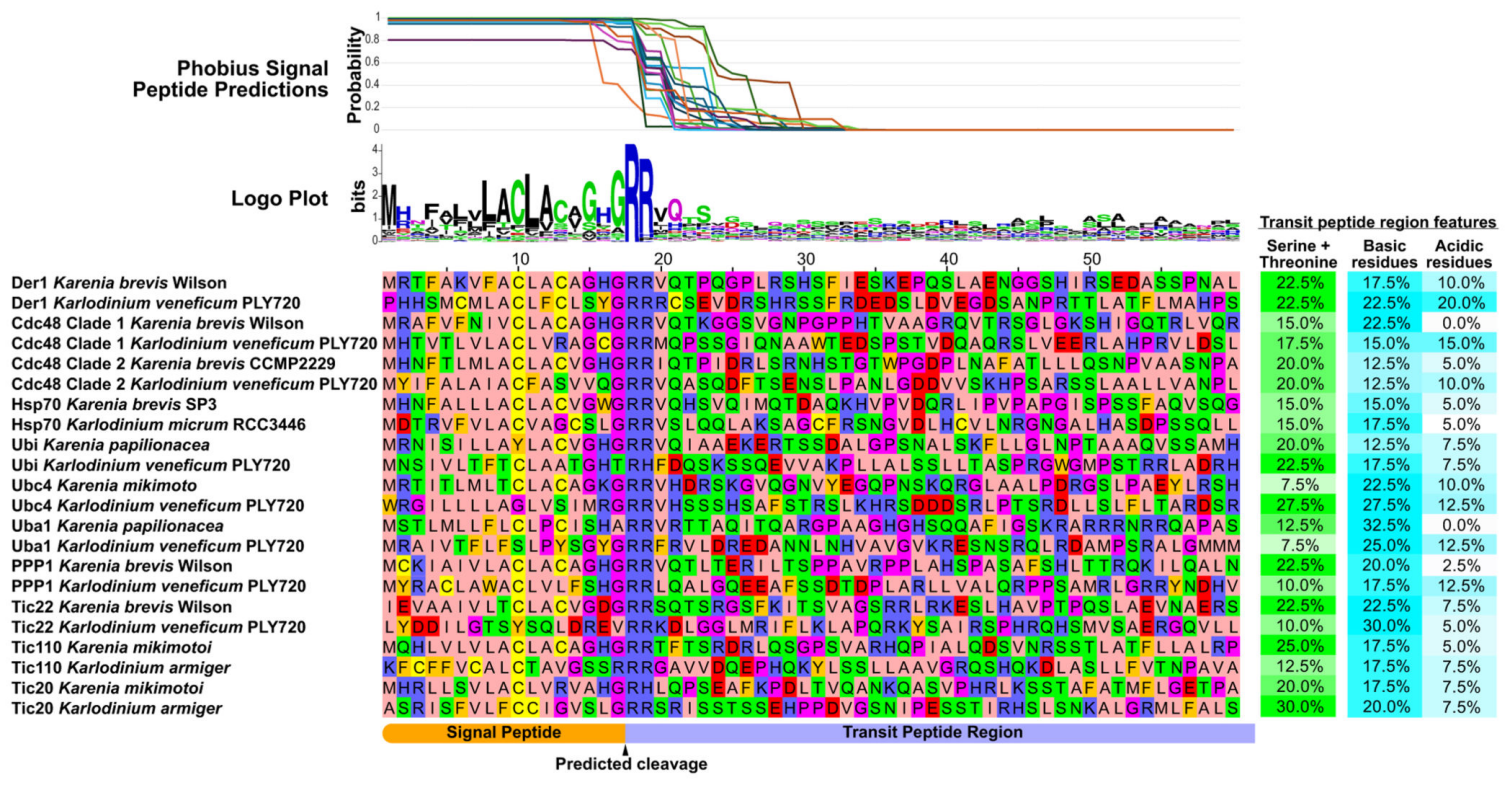
Haptophyte-derived Kareniaceae translocon machinery possess bipartite plastid-targeting presequences ER-directing signal peptide (SP) predictions by Phobius^32^ for 20 Kareniaceae translocation components. A logo plot made using WebLogo^33^ of protein pre-sequences aligned on the predicted cleavage site. Alignment of protein presequences with transit peptide-type features (40 residues post-SP cleavage site) is shown for each.

The plastids known to use SELMA at the third membrane—in haptophytes, cryptophytes, ochrophytes, and apicomplexans—are surrounded by a fourth, final bounding membrane. This outer membrane is part of the cell’s endomembrane network and either shares direct continuity with the ER or is connected to it by vesicle trafficking.^5^ Therefore, the first step of protein delivery to these plastids is the cotranslational import of proteins into the ER. Proteins for such plastids bear an N-terminal signal peptide for recognition and delivery into the ER through the Sec61 complex.^31^ This signal peptide is then typically proteolytically removed, and a downstream protein-sorting “transit peptide” is responsible for subsequent recognition and sorting through SELMA, TOC, then TIC. To test if the haptophyte-derived SELMA proteins in the Kareniaceae are likely still deployed to and used in plastid import, rather than having been co-opted by its cytosol-orientated ERAD, we examined these proteins for evidence of the bipartite plastid-targeting signals. All of these SELMA proteins had recognizable signal peptides followed by a further protein extension with the typical features of transit peptides—elevated serine/threonine content and enrichment for basic over acidic residues particularly at the N terminus (Figure 4). These features were also seen for the TOC/TIC proteins, and together they support that the haptophyte-derived translocons are coded for by nuclear genes and delivered to and function in the plastid.

### Serial gain of haptophyte plastids in the Kareniaceae did not add further membranes

**Figure 5:**
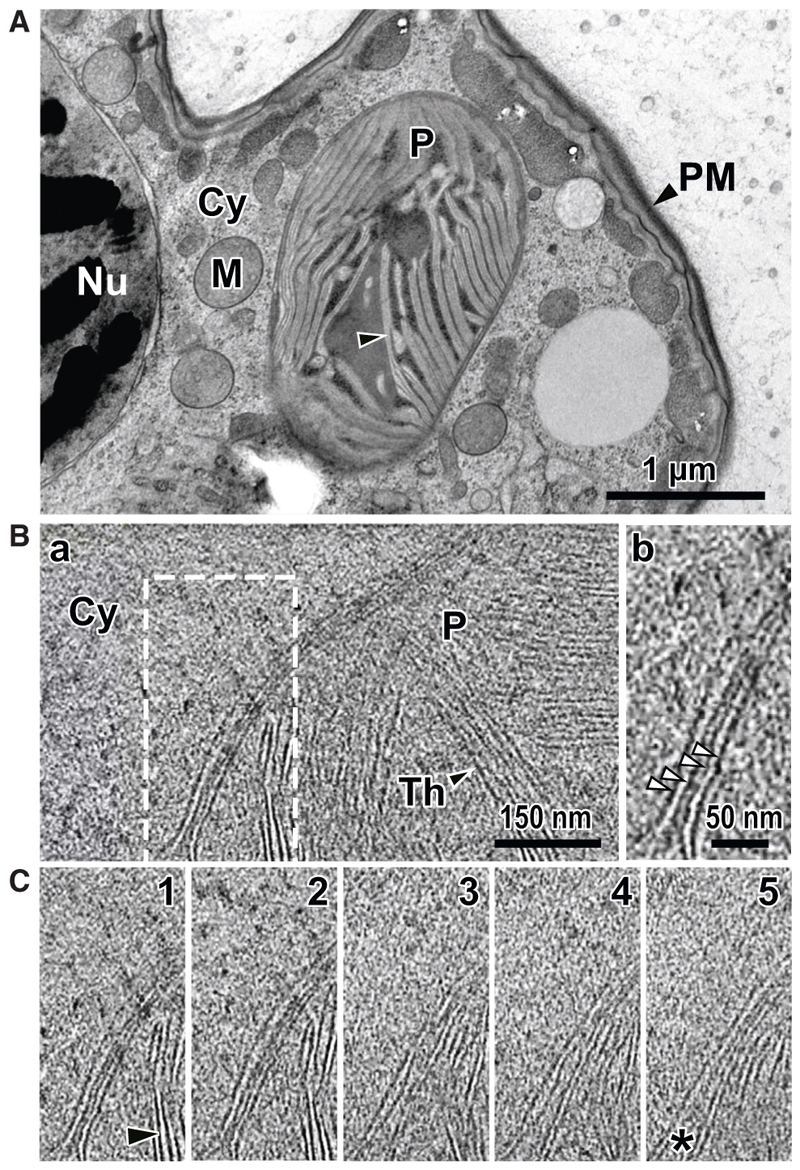
*Karlodinium veneficum* plastids are surrounded by four membranes (A) Transmission electron micrograph of a high-pressure frozen, resin-embedded, thin section of the cell periphery showing a plastid (P) within the cytoplasm (Cy). (B) Cryo-electron tomogram showing the membranes separating the plastid thylakoids (Th, black arrowhead) and stroma from the cytoplasm. White dashed boxed region is magnified in (B) where white arrowheads indicate four bounding membranes of the plastid. (C) Z series of five successive virtual sections of the tomogram area shown by the dashed box in (B). The bounding membranes show the outer and inner membrane pairs maintaining an approximately fixed separation distance between each membrane, but variation in the spacing between these pairs is seen (asterisk). Nu, nucleus; M, mitochondrion; Cy, cytoplasm; P, plastid; PM, plasma membrane; Th and black arrowheads, thylakoids.

Our analysis of translocons in the Kareniaceae dinoflagellates accounts for a possible four membranes surrounding these plastids. However, endosymbiotic processes are often assumed to entail the gain of further membranes, presumed to typically derive from the enveloping phagolysosomal membrane and the endosymbiont’s own plasma membrane, although other scenarios of membrane gain have been proposed.^34^ Such acquired, further membranes would presumably require additional protein translocons, which might present further obstacles to endosymbiont establishment. The number of membranes surrounding the plastids of *Karlodinium* and *Karenia* has been previously unclear, with transmission electron microscopy (TEM) of resin-embedded specimens consistently showing poor plastid membrane preservation and collapsed membrane profiles (Figure 5A). To overcome this, we used cryo-electron tomography (cryoET) on flash frozen *Karlodinium veneficum* cells prepared as thin lamellae cell sections by focused-ion beam (FIB) milling. Reconstructed tomograms of these sections clearly distinguished the separate bounding plastid membranes from the thylakoid membranes and showed the number of bounding membranes to be four (Figures 5B and 5C). These data show that the acquisition of the haptophyte plastid in Kareniaceae dinoflagellates did not result in additional bounding membranes; therefore, the haptophyte-derived translocons alone are likely sufficient for protein import.

### The ancestral dinoflagellate peridinin plastid lacks SELMA, and this plastid likely persists in Kareniaceae with haptophyte plastids

The ancestral “peridinin” plastid of dinoflagellates is surrounded by only three membranes, which distinguishes it from the four-membrane-bound plastids of most other organisms with complex plastids.^35^ In none of our phylogenetic analyses were SELMA orthologs found in dinoflagellates that only contain this ancestral plastid, with 45 species sampled across 30 genera (Figures 1, 2, and 3; Data S1). The ERAD paralogs of the ER, on the other hand, were ubiquitously present in these taxa, showing that available sequence coverage was not limiting these searches. Furthermore, our analyses show that in the related api-complexans, even the basal groups with a plastid retain SELMA proteins (e.g., the marosporidian *Rhytidocystis*, gregarines *Selendinium* and *Siedleckia*, chromopodellid *Piridium*, and squirmid *Digyalum*), whereas taxa that have lost the plastid also lack SELMA orthologs (*Cryptosporidium* spp. and gregarines *Cephaloidophora* and *Heliospora*) (Figures 2 and 3; Data S1). In the dinoflagellates with the peridinin plastid, orthologs of Tic110, Tic20, Tic22, and PPP1 were consistently found (Figure 1), further implicating PPP1 as part of the TOC and indicating that this plastid likely only requires the canonical TOC/TIC for protein translocation across the inner membranes. Vesicular fusion from the endomembrane system is known to deliver proteins across the outermost membrane in peridinin plastids.^36^ Whether SELMA occurred previously in dinoflagellates but was lost along with the third membrane, or was never present with this plastid, is unknown.

**Figure 6:**
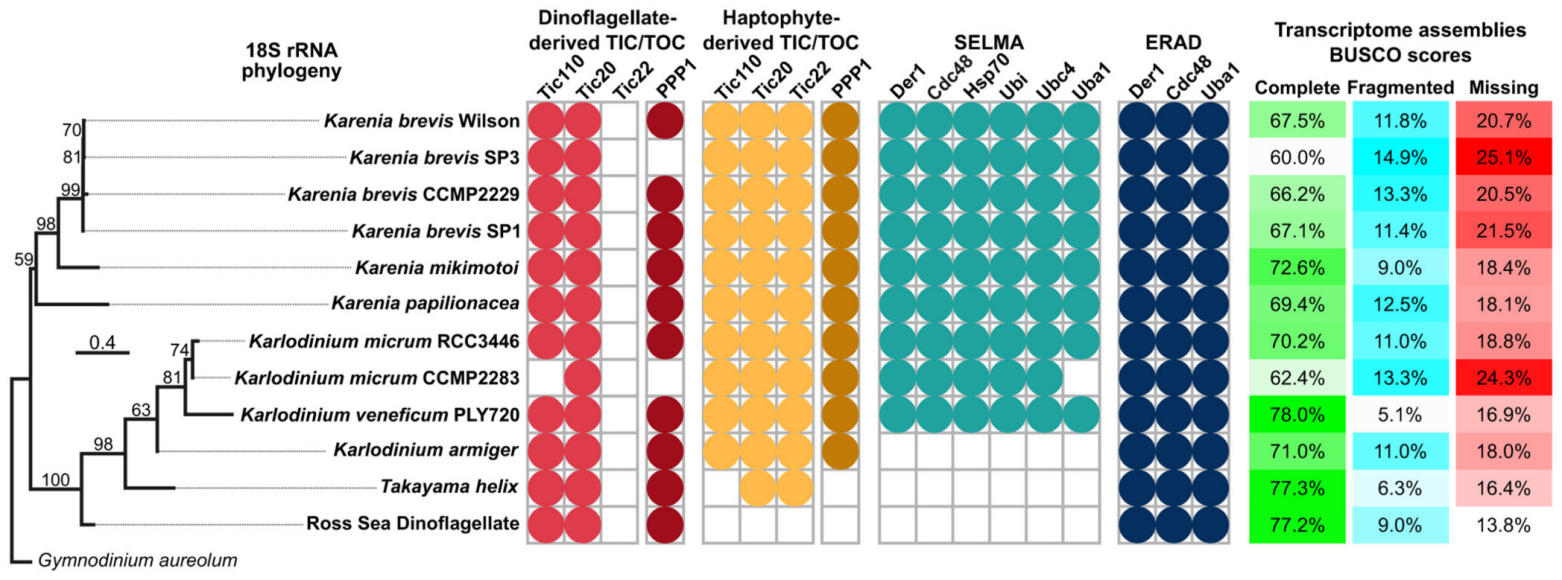
Presence and absence of plastid translocons in Kareniaceae Phylogeny of Kareniaceae dinoflagellates and the detection of expressed plastid translocon proteins in each taxon indicated by colored circles. BUSCO scores, as an estimate of transcriptome coverage, are given for each. Bootstrap support values for phylogeny nodes are shown, and the scale bar indicates estimated number of nucleotide substitutions per site.

Unexpectedly in Kareniaceae, peridinin plastid orthologs of Tic110, Tic20, and PPP1 were found in addition to orthologs derived from haptophytes (Figures 1B, 1C, 1E, and 6). These data suggest the presence of two phylogenetically distinct plastids in Kareniaceae where a cryptic relic of the peridinin plastid has been retained despite the presence of the new haptophyte-derived plastids for photosynthesis. It is conceivable that these peridinin-type translocons could function now in the haptophyte-derived plastids as duplicates of the import machinery. However, these translocons are also present in other dinoflagellates with non-photosynthetic relic peridinin plastids (Kryp-toperidiniaceae and *Crypthecodinium conhii*, Data S1)^37,38^ and are exclusively expressed in the RSD where haptophyte versions are not expressed (Figure 6), collectively indicating that these peridinin-type translocons serve as markers for the persistence of these original peridinin plastids. Direct evidence of this relict organelle in Kareniaceae is now required.

### SELMA loss has occurred in instances of further plastid replacement in Kareniaceae

In two Kareniaceae species, further instances of plastid gain have occurred in apparent like-for-like plastid replacements. Phylogenies of plastid-encoded genes show that *Karlodinium armiger* and *Takayama helix* have plastids more closely related to *Prymnesium* and *Phaeocystis* haptophytes, respectively, than to those of the other *Karlodinium* or *Karenia* spp.^20^ In both cases, previously acquired nucleus-encoded haptophyte-derived genes apparently continue to service these new plastids.^20^ This indicates that many or all nucleus-encoded plastid genes were compatible with the replacement plastids when gained from closely related groups (i.e., other haptophytes) and that these genes provide a preadaptation for possible ongoing plastid replacements. Indeed, we see in *K. armiger* that even the TICs for protein import are of common origin with the other *Karlodinium* spp. and therefore have also facilitated plastid replacement (Data S1). Surprisingly, both *K. armiger* and *T. helix* lack any genes for SELMA (Figure 6; Data S1). These losses of the SELMA complex mean that the translocon complement of *K. armiger* and *T. helix* resembles that of the peridinin plastid that is surrounded by only three membranes. This suggests that the acquisition of new plastids in both *K. armiger* and *T. helix* might have involved the elimination of one bounding membrane. Bipartite plastid-targeting sequences are used for both three- and four-membrane plastids.^31^ Therefore, the loss of the equivalent of the third (SELMA) membrane would not be predicted to disrupt protein import of existing nucleus-encoded proteins.

A further Kareniaceae species, the RSD lacks a stable haptophyte-derived plastid but does acquire and maintain temporary haptophyte endosymbionts captured from *Phaeocystis* spp.^16^ Although some haptophyte genes occur in the nucleus of this dinoflagellate,^17^ we detected no expression of haptophyte SELMA or even haptophyte TIC proteins in their transcriptomes (Figure 6). Thus, any protein import in this system must rely on the translocons acquired *in situ* with the “kleptoplast,” and they would seemingly not be able to be resynthesized if damaged or degraded.

### Dinoflagellates with additional paralogous ERAD machineries

A different group of dinoflagellates (family Kryptoperidiniaceae of the Peridiniales) have also acquired new alternative plastids, in this case derived from diatom endosymbionts. These so-called “dinotom” dinoflagellates stably maintain, replicate, and inherit these new endosymbionts.^14^ Dinotoms are unusual, however, in that a further single membrane is present surrounding their endosymbionts creating a fifth membrane separating the plastid lumen from host cytosol.^39^ No protein targeting from the host cell to endosymbiont has apparently been established.^40^ Thus, these endosymbionts maintain a diatom nucleus that encodes and expresses most plastid-related proteins that then translocate across the existing four diatom membranes surrounding the stroma and thylakoids. Given the relative intactness of the diatom as an endosymbiont, we hypothesized that the diatom SELMA machinery would have been retained to enable protein targeting of the diatom-encoded plastid proteins. Our phylogenies of all the SELMA proteins, as well as the TIC proteins, showed that ochrophyte-grouping orthologs for plastid import are all present in the dinotoms (*Kryptoperidinium triquetrum* and *Glenodinium foliaceum*) consistent with maintenance of the diatom-derived machinery (Figures 1, 2, and 3; Data S1). Dinotoms also possess the ERAD proteins that group with the dinoflagellate ERAD orthologs as expected for the presence of the canonical ER machinery. However, an additional set of ERAD paralogs is also present in dinotoms, and these proteins group with the diatom/ochrophyte ERAD proteins (Figures 1, 2, and 3; Data S1). These data indicate that the ER of the diatom symbiont still retains its ERAD protein quality control processes. Thus, dinotoms maintain three paralogous ERAD machineries—two serving two functional but separate ER systems and one (SELMA) representing the repurposed plastid translocon—and provide a second example of ERAD duplication through endosymbiosis.

### Kareniaceae present a model for serial red plastid acquisition and exchange

**Figure 7:**
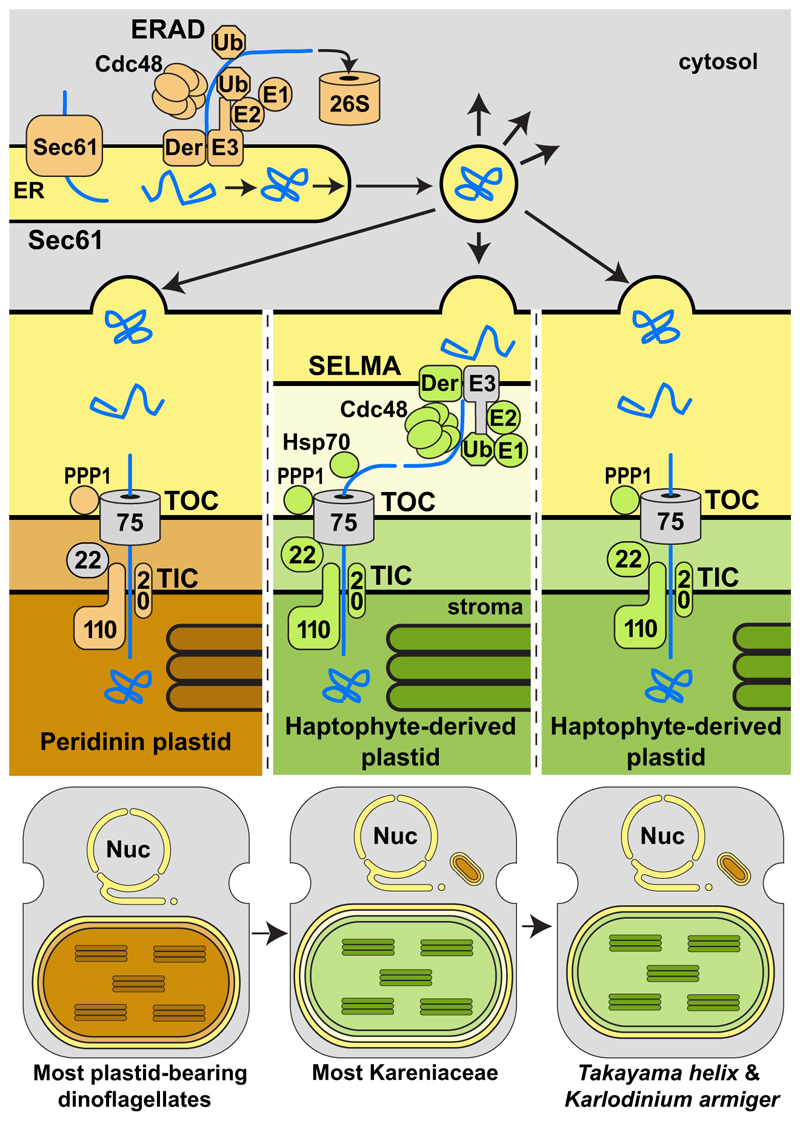
Model of ancestry and evolution of plastid-protein translocation machinery in Kareniaceae dinoflagellates Ancestral peridinin plastids of dinoflagellates are surrounded by three membranes, and protein targeting occurs via the ER translocon Sec61 and vesicle delivery. Upon outer membrane fusion, protein cargo then passes through the TOC and TIC complexes. Most known Kareniaceae dinoflagellates inherited new plastids derived from haptophytes and likely adopted the dinoflagellate routing through the ER to the outermost plastid membrane. From here, haptophyte-derived translocons (green), including SELMA, complete protein import. Further plastid replacement in *T. helix* and *K. armiger* with alterative haptophyte plastids has maintained the preexisting TICs but eliminated all SELMA components and presumably the equivalent plastid membrane. Gray translocons remain unidentified.

This study shows that plastids in the Kareniaceae dinoflagellates have not accrued further membranes during their acquisition from haptophytes and that these plastids maintained all the necessary translocons from the source haptophytes for protein import (Figure 7). Effectively, these plastid organelles occur and function in the cytosol of Kareniaceae dinoflagellates as they would have previously in the cytosol of haptophytes. Most genes for haptophyte-derived plastid proteins are known to have been transferred to the dinoflagellate nuclei,^20,40–43^ and given that they would have possessed preexisting bipartite leaders for targeting to haptophyte plastids, their expression in the new host could facilitate protein uptake into this plastid as before. The sorting of plastid proteins within the ER/endomembrane system to target them to the plastid would be the only necessary adaptation required after the acquisition of these new plastids, and it is hard to predict how difficult this might be to develop. But in the Kareniaceae, this process might have also exploited existing protein-sorting routes used for proteins of the relict peridinin plastid that our data suggest still cooccurs today. The discrimination of proteins required in the two different plastids might have been assisted through the SELMA-based selection of proteins for the haptophyte plastid that is absent for the peridinin plastid. In any case, it is apparent that little or no new translocation machinery needed to be developed for a preexisting complex red-type plastid to be transferred to a different eukaryotic lineage.

This model for plastid exchange presented by the Kareniaceae suggests that an intact haptophyte algal endosymbiont likely never occurred as an intermediate stage in their evolution, unlike that seen for the seemingly stalled diatom endosymbionts of Kryptoperidiniaceae dinotoms. In dinotoms, the additional (fifth) membrane around the diatom symbiont might present one protein trafficking challenge too many for the development of a protein import process.^44^ The persistence of diatom nuclei, ERAD, and SELMA indicates that dinotom plastid proteins still co-translationally enter the diatom ER *en route* to the plastid. This process is likely incompatible with any further preceding trafficking events across the outermost fifth membrane and from the dinoflagellate cytosol. A consequence of the model of direct organelle procurement inferred for Kareniaceae is that the transfer of plastid genes to the nucleus of the new host would have to occur by way of repeated feeding events on the same or closely related algal taxa rather than from a constantly maintained symbiont nucleus. The RSD and the euglenid *Rapaza* provide examples of this process where temporary kleptoplasts receive some maintenance from small numbers of genes acquired in the host’s nucleus by horizontal gene transfer from their prey’s nuclei.^17,45^ This process has seemingly continued to completion in the Kareniaceae where the once kleptoplasts are now permanent stable organelles with no persistent haptophyte nuclei.

A corollary of the protracted process of endosymbiont gene gain is that a long history of constant organelle gain and turnover would have occurred. Indeed, there is evidence that *Karlodinium* and *Karenia* contain similar, but taxonomically distinct, haptophyte plastids, suggesting that they ultimately fixed different stable endosymbionts.^16,20^ *K. armiger* and *T. helix* provide further evidence of the ongoing transmissibility of complex red plastids, each possessing a more recently replaced version of a haptophyte-derived plastid.^16,20^ Kareniaceae dinoflagellates remain eukaryovorous, sucking up prey organelles by myzocytotic feeding, and it is plausible that further cases of plastid replacement will be identified in this group.^46^ It is curious that *K. armiger* and *T. helix* both lost SELMA with their plastid replacements, and presumably the third membrane which SELMA is required to translocate proteins across. This apparent reversion to the state of the peridinin plastid raises the question of whether the canonical dinoflagellate three-membrane-bound plastid is an equivalent replacement of a possible ancestral SELMA-containing four-membrane plastid such as that found throughout the sister lineage, Apicomplexa.

In principle, we see no reason why the model of complex red plastid gain in the Kareniaceae might not also account for the gains of equivalent plastids in haptophytes, ochrophytes, and apicomplexans and perhaps even separate gains within these groups, including in chrompodellids *Chromera* and *Vitrella*. In all cases the same number of membranes surround the plastid, and the same translocons and trafficking steps occur in all.^26,31,34,47^ The possible number of exchanges of serial secondary plastids and their directions of travel might be difficult to gauge. But this model provides a mechanistic solution to the “Rhodoplex” hypothesis,^8^ which posits the gain of much of aquatic photosynthetic diversity through multiple exchanges of a single red-algal-derived secondary plastid,^6,9–11,48,49^ as an alternative to the now phylogenetically impossible single ancestral gain predicted by the original chromalveolate hypothesis owing to the common ancestor of chromalveolate lineages now being recognized as older than red algae themselves.^6,9–11,48,49^

## Resource Availability

### Lead contact

Further information and requests for resources should be directed to and will be fulfilled by the lead contact, Ross Waller (rfw26@cam.ac.uk).

### Materials availability

This study did not generate new unique reagents.

### Star★Methods

Detailed methods are provided in the online version of this paper and include the following:

- KEY RESOURCES TABLE
- EXPERIMENTAL MODEL AND STUDY PARTICIPANT DETAILS
  ○ Cultivation of *Karlodinium veneficum* PLY720
- METHOD DETAILS
  ○ RNA extraction
  ○ Library preparation, sequencing, transcriptome assembly and gene prediction
  ○ BUSCO analysis
  ○ Database sampling and phylogenetic analysis
  ○ Sample preparation for transmission electron microscopy
  ○ Cryo-FIB lamella preparation, cryo-ET data collection and processing
- QUANTIFICATION AND STATISTICAL ANALYSIS

## Star★Methods

### Key Resources Table

**Table:** 

REAGENT or RESOURCE	SOURCE	IDENTIFIER
Experimental model: Organism/strain		
*Karlodinium veneficum* PLY720	Plymouth Culture Collection of Marine Algae, Marine Biological Association, Plymouth, UK	PLY720
Chemicals		
TRIzol	Thermo Fisher Scientific	15596026
Critical commercial assays		
PacBio Iso-Seq Express Template Preparation Kit	PacBio	N/A
PacBio Sequel II SMRT cell	PacBio	N/A
Illumina Stranded mRNA Prep Kit	Illumina	N/A
Deposited data		
Raw sequencing data	This paper	NCBI BioProject: PRJNA1117636
Assembled transcriptome data and predicted proteins	This paper	Figshare: https://figshare.com/s/41c2e3c38d039359816c, DOI: https://doi.org/10.6084/m9.figshare.21602697
UniProt	-	https://www.uniprot.org/
MMETSP	Keeling et al.^50^	https://doi.org/10.5281/zenodo.3247846
VEuPathDB	Alvarez-Jarreta et al.^51^	https://veupathdb.org/
Kareniaceae transcriptomes	Novák Vanclová et al.^20^	N/A
Apicomplexa transcriptomes	Mathur et al.^52^	N/A
Apicomplexa transcriptomes	Janouškovec et al.^53^	N/A
Software and algorithms		
Trinity version 2.11.0	Grabherr et al.^54^	https://github.com/trinityrnaseq/trinityrnaseq/releases/tag/v2.11.0
TransDecoder version 5.5.0	-	https://github.com/TransDecoder/TransDecoder/releases/tag/TransDecoder-v5.5.0
Busco version 5.5.0	Manni et al.^55^	https://gitlab.com/ezlab/busco/-/releases/5.5.0
AlphaFold2	ColabFold v1.5.5	https://github.com/sokrypton/ColabFold
NCBI-VAST	Madej et al.^56^	https://www.ncbi.nlm.nih.gov/Structure/VAST/vastsearch.html
Foldseek	van Kempen et al.^57^	https://search.foldseek.com/search
CD-HIT version 4.8.1	Fu et al.^58^	https://github.com/weizhongli/cdhit/releases/tag/V4.8.1
MAFFT version 7.475	Katoh et al.^59^	https://mafft.cbrc.jp/alignment/software/mafft-7.475-with-extensions-src.tgz
trimAl version 1.4	Capella-Gutiérrez et al.^60^	https://github.com/inab/trimal/releases/tag/v1.4.1
FastTree version 2.1.11	Price et al.^61^	www.microbesonline.org/fasttree/
IQ-TREE version 2.1.2	Price et al.^61^	https://github.com/iqtree/iqtree2/releases/tag/v2.1.2
UFBoot2	Hoang et al.^62^	https://github.com/iqtree/iqtree2/releases/tag/v2.1.2
ModelFinder	Kalyaanamoorthy et al.^63^	https://github.com/iqtree/iqtree2/releases/tag/v2.1.2
Barrnap version 0.9		https://github.com/tseemann/barrnap/releases/tag/0.9
WARP version 1.0.9	Tegunov et al.^64^	N/A
AreTomo	Zheng et al.^65^	N/A

### Experimental Model and Study Participant Details

#### Cultivation of *Karlodinium veneficum* PLY720

*Karlodinium veneficum* PLY720 was obtained from The Plymouth Culture Collection of Marine Algae, Marine Biological Association, Plymouth, UK. The culture collection records report that this strain was originally isolated in 1976 from a marine sample collected from a fjord in Norway (59°30^′^N 10°36^′^E), and that it is synonymous with the strains CCMP415 held at the National Center for Marine Algae and Microbiota, Bigelow, USA, and NEPCC 734 held at The Canadian Center for the Culture of Microorganisms, Vancouver, Canada. Cells were grown in L1 medium, typically in 200 ml volumes in T175 culture flasks. Culture flasks were maintained in an incubator with a 14:10 hours light/dark cycle, using a light intensity of approximately 20 μmol m^-2^ s^-1^ provided by LEDs incubated at a consistent temperature of 20 °C and sub-cultured monthly by inoculating filter-sterilised L1 medium with mature culture, typically in a volume ratio of 40:1.

## Method Details

### RNA extraction

To prepare samples for long-read sequencing, cells from two 200 ml *Karlodinium veneficum* PLY720 cultures were harvested; one six hours into the light phase and one four hours into the dark phase of a culture light/dark cycle. Samples for short-read sequencing were prepared as part of a concurrent unpublished study in which cells from twenty-one 200 ml *Karlodinium veneficum* PLY720 cultures were harvested. In all cases cells from these 200 ml cultures were collected by decanting the cultures into four 50 ml tubes, which were then centrifuged at 5000 x g for 5 minutes at 4 °C. The supernatants were then discarded and the pellets pooled by transferring to a 2 ml microcentrifuge tube. This tube was then centrifuged using the same conditions as the first centrifugation, after which the supernatant was discarded and the tube containing the remaining cell pellet was flash-frozen in liquid N_2_ and then stored at -80 °C until further processing. All samples for both long and short read sequencing were then processed in the same way to extract RNA using TRIzol, with a detailed step-by-step description of the methods deposited at protocols.io (https://doi.org/10.17504/protocols.io.n92ld9xe7g5b/v1).

### Library preparation, sequencing, transcriptome assembly and gene prediction

PacBio long-read sequencing was performed by the Earlham Institute, Norwich, UK. This included the preparation of sequencing libraries from the extracted RNA using the PacBio Iso-Seq Express Template Preparation (v2, no size-selection) kit, sequencing the libraries using a PacBio Sequel II SMRT cell (8M, v2, 30hr Movie), and performing IsoSeq3 analysis to process and refine the raw reads and generate HiFi reads. Illumina short read sequencing was performed by the Genomics department at Cancer Research UK Cambridge Institute. This included preparation of sequencing libraries using the Illumina Stranded mRNA Prep Kit, which were then sequenced using an Illumina NovaSeq 6000 system with a SP flowcell to generate 2 x 50 bp (paired-end) reads. Long reads and short reads were co-assembled using Trinity version 2.11.0^54^ and gene prediction performed using TransDecoder version 5.5.0 (https://github.com/TransDecoder/TransDecoder/releases/tag/TransDecoder-v5.5.0).

### BUSCO analysis

BUSCO analysis was performed on predicted peptide files for all twelve Kareniaceae transcriptomes investigated in the present study using Busco version 5.5.0^55^ with the eukaryota_odb10 busco reference dataset.

### Database sampling and phylogenetic analysis

A detailed description of the methodology and workflow, including the code used to perform the phylogenetic analyses for translocon proteins is deposited at GitHub (https://github.com/camwallerlab/Methods-for-phylogenetic-analysis-of-plastid-translocons). Briefly, this involved creating a custom protein sequence database from the datasets listed in Table S1 obtained from UniProt, the MMETSP reassemblies (https://doi.org/10.5281/zenodo.3247846),^50^ and VEuPathDB,^51^ as well as transcriptomes for organisms sequenced in three previous studies^20,52,53^ and the *Karlodinium veneficum* PLY720 transcriptome generated in the present study. This custom database was then searched using blastp (BLAST+ version 2.11.0) for homologues of each of the translocon proteins investigated in the present study using characterised versions of these proteins as queries. For Toc75, where blastp did not recover any putative orthologues in the Kareniaceae or other dinoflagellates, we also created Hidden Markov model (HMM) with known Toc75s for profile searches using HMMER (hmmer.org). While no significant matches were recovered, the structures of the best matches were modelled with AlphaFold2 via ColabFold v1.5.5 and these were assessed for similarity to known Toc75 structures using NCBI-VAST^56^ and Foldseek.^57^ No credible Toc75 candidates were recovered by these methods either. The homologues obtained from these searches were then clustered with cdhit (CD-HIT version 4.8.1)^58^ to remove highly similar sequences, thereby reducing overall redundancy of the dataset. Iterative rounds of alignment using mafft (MAFFT version 7.475),^59^ conserved site selection using trimal (trimAl version 1.4),^60^ tree inference with FastTreeMP (FastTree version 2.1.11)^61^ using the default settings and manual removal of sequences were then performed. The purpose of these iterations was to further reduce dataset redundancy, as well as to identify and remove dissimilar or poorly aligning sequences. The final curated datasets for each translocon protein that these methods obtained were then aligned using mafft-linsi (MAFFT version 7.475) and conserved sites were selected using trimal (trimAl version 1.4). A phylogeny was then inferred for each translocon dataset using the program iqtree2 (IQ-TREE version 2.1.2)^61^ with 1000 ultrafast bootstrap replicates (UFBoot2)^62^ and using the best-fitting model, LG+F+I+G4^66,67^ that was chosen according to the Bayesian Information Criterion by ModelFinder,^63^ all implemented within iqtree2.

For the 18S rRNA gene phylogeny, nucleotide sequences for each of the Kareniaceae species studied were extracted from the corresponding transcriptome assemblies using barrnap (Barrnap version 0.9, https://github.com/tseemann/barrnap). Sequences were aligned using mafft-linsi (MAFFT version 7.475) and conserved sites were selected using trimal (trimAl version 1.4). A tree was then inferred from the trimmed alignment using the program iqtree2 (IQ-TREE version 2.1.2) with 1000 ultrafast bootstrap replicates and using the best-fitting model, TN+F+I,^68^ that was chosen according to the Bayesian Information Criterion by ModelFinder, all implemented within iqtree2.

### Sample preparation for transmission electron microscopy

High-pressure freezing (HPM100, Leica) followed by freeze substitution (EM ASF2, Leica) was conducted to prepare samples for electron microscopy following published protocols.^69^ Cultured *Karlodinium veneficum* PLY720 cells were harvested at exponential growth phase by centrifugation at 2500 x for 2 minutes prior to cryo-fixation with high pressure freezing. Resin blocks were obtained after the freeze substitution.^69^ For TEM analysis, ultrathin sections of 60 nm thickness were mounted onto copper grids or slots coated with formvar and carbon. Sections were then stained in 1% uranyl acetate (10 min) and lead citrate (5 min). Micrographs were obtained using a Tecnai G2 Spirit BioTwin microscope (FEI) operating at 120 kV with an Orius SC1000 CCD camera (Gatan).

### Cryo-FIB lamella preparation, cryo-ET data collection and processing

*Karlodinium veneficum* PLY720 cells were allowed to settle in a 10 μl aliquot upon a poly-L-lysine coated holey carbon film gold grid that was previously glow-discharged for 60 s. Grids were sealed in a plastic container and transferred to a 20 °C incubator and after 2 hrs, a second aliquot was added and incubated again. Before vitrification, grids were manually blotted on the reverse side for 2 s, then plunged into liquid ethane with an FEI Vitrobot (100% humidity) and stored in liquid nitrogen until used. Grids were first screened in a Talos Arctica operated at 200 kV (equipped with a Falcon 3EC detector) and grids of good quality were chosen for FIB-milling. For milling, the grid was transferred to a Zeiss crossbeam 550 Gemini 2 system and a layer of platinum was sputtered to the surface of sample for 1 min 20 s. Rough milling was performed automatically at a current range of 700-100 pA, and polishing was performed manually at 50 pA, aiming for a lamella thickness of 180 nm. An additional Pt layer was added to stabilise the lamellae for 2 seconds at 5 mA.

The thin lamellae were imaged on a Titan Krios G2 transmission electron microscope (Thermo Fisher Scientific/FEI) operated at 300 kV equipped with a Gatan K3 direct electron detector. Tilt series were acquired with a dose-symmetric scheme, with a 3º increment between +60º and -60º. A pixel size of 3.5 Å /pixel was used, with a defocus range from -3.0 to -5.0. Images were acquired with a total dose of 73.4 e^−^ (Å) ^−2^. Gain-correction, motion correction and defocus estimation were performed in WARP 1.0.9.^64^ The tilt series were aligned in AreTomo,^65^ and tomograms were reconstructed in AreTomo with a binning of 8 (28 Å/pixel) and using simultaneous algebraic reconstruction technique (SART).^70^

## Quantification and Statistical Analysis

All phylogenetic analyses (Figures 1, 2, 3, and 6) were performed using iqtree2 (IQ-TREE version 2.1.2)^61^, with 1000 ultrafast boot-strap replicates (UFBoot2)^62^ and using the best-fitting model for each (LG+F+I+G4^66,67^ for protein phylogenies, TN+F+I^68^ for the 18S rRNA phylogeny), which were identified by ModelFinder using the Bayesian Information Criterion, all implemented within iqtree2.

## Supplementary Material

Data S1, related to Figures 1, 2, and 3.

Document S1. Table S1, related to Figures 1, 2, and 3.

## Data Availability

Accession numbers for the sequences used in the phylogenetic analyses can be found in Data S1, and the sources of these sequences are given in Table S1. Raw sequencing data used to assemble the Karlodinium veneficum PLY720 reference transcriptome are available for download from the NCBI Sequence Read Archive: PRJNA1117636 (BioSample accession SAMN41577327). Assembled transcriptome data, including transcripts and predicted proteins in fasta format, in addition to files that were used to generate phylogenies, including unaligned sequences, aligned sequences, trimmed alignments, and output files from IQ-TREE 2, are available at Figshare (https://figshare.com/s/41c2e3c38d039359816c, https://doi.org/10.6084/m9.figshare.21602697).

## Highlights

- Plastid-protein translocons can be inherited with plastid transfers between organisms
- Dinoflagellate *Karlodinium veneficum* plastids have only four bounding membranes
- Four bounding membranes are not indicative of secondary endosymbioses only
- Serial plastid replacement utilizes existing protein import systems

## In brief

Lewis et al. use dinoflagellate algae that have replaced their photosynthetic plastid organelles to investigate the processes of organelle establishment. They show that additional membranes are not always gained in serial endosymbioses and that key features of plastid-protein-targeting and maintenance can be maintained from preexisting symbioses.
