# Supplementary figures and images for: Plastid translocon recycling in dinoflagellates demonstrates the portability of complex plastids between hosts
Source: Curr Biol. Author manuscript; Available in PMC 2025 Feb 25. (PMC7617431; doi:10.1016/j.cub.2024.10.034)

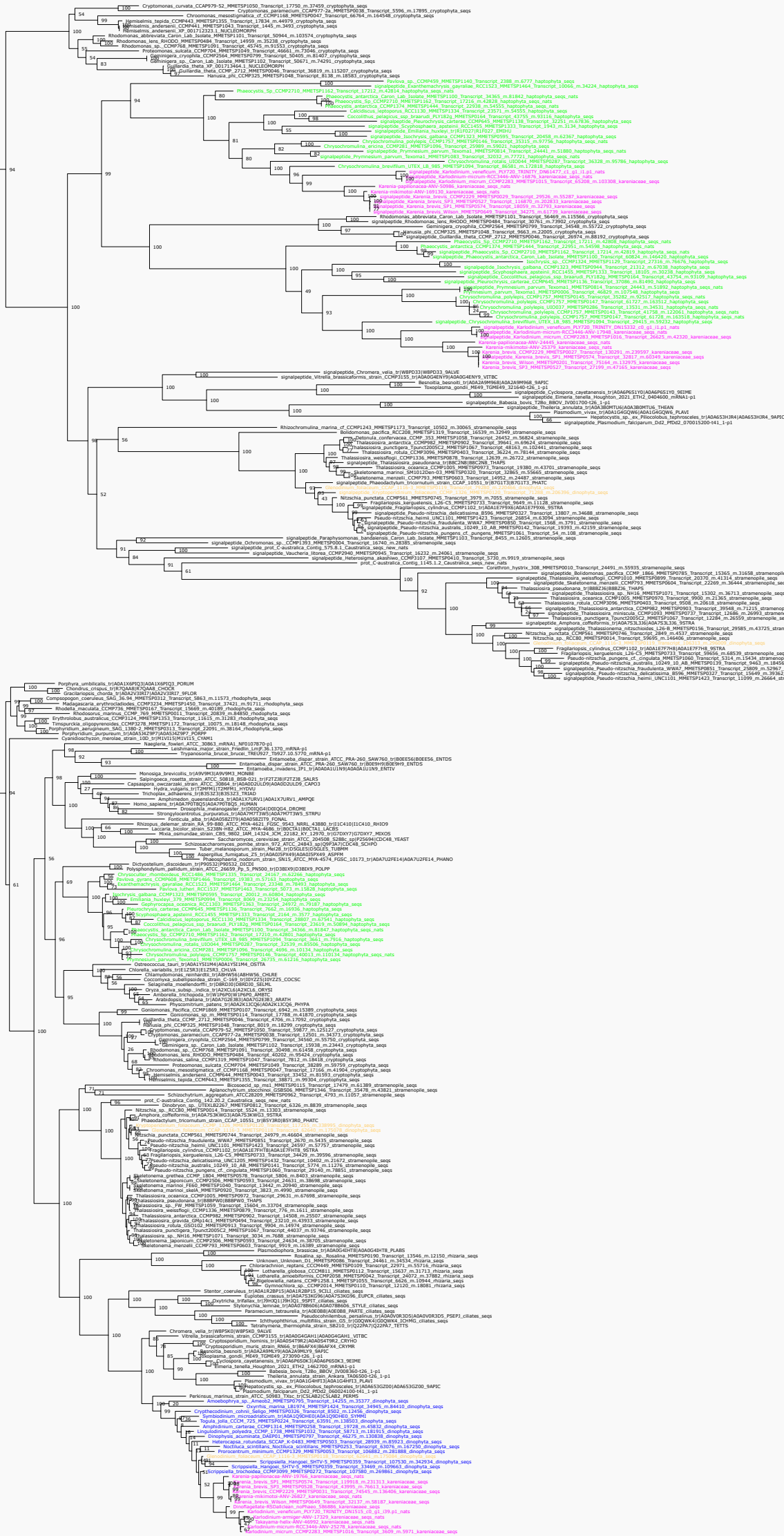

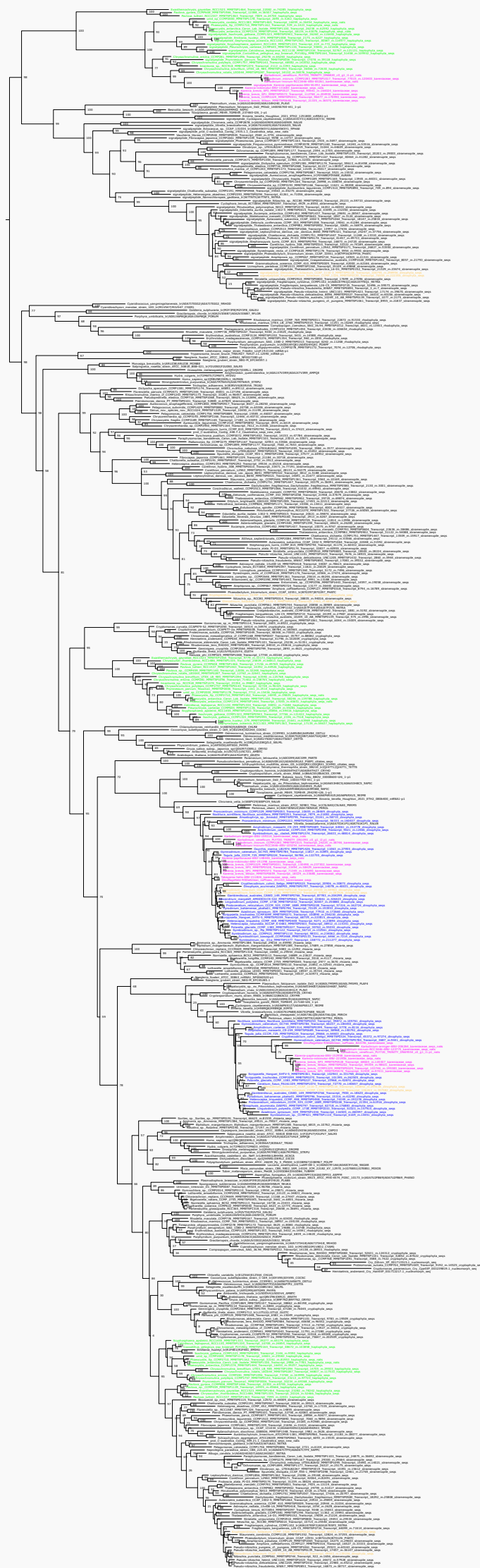

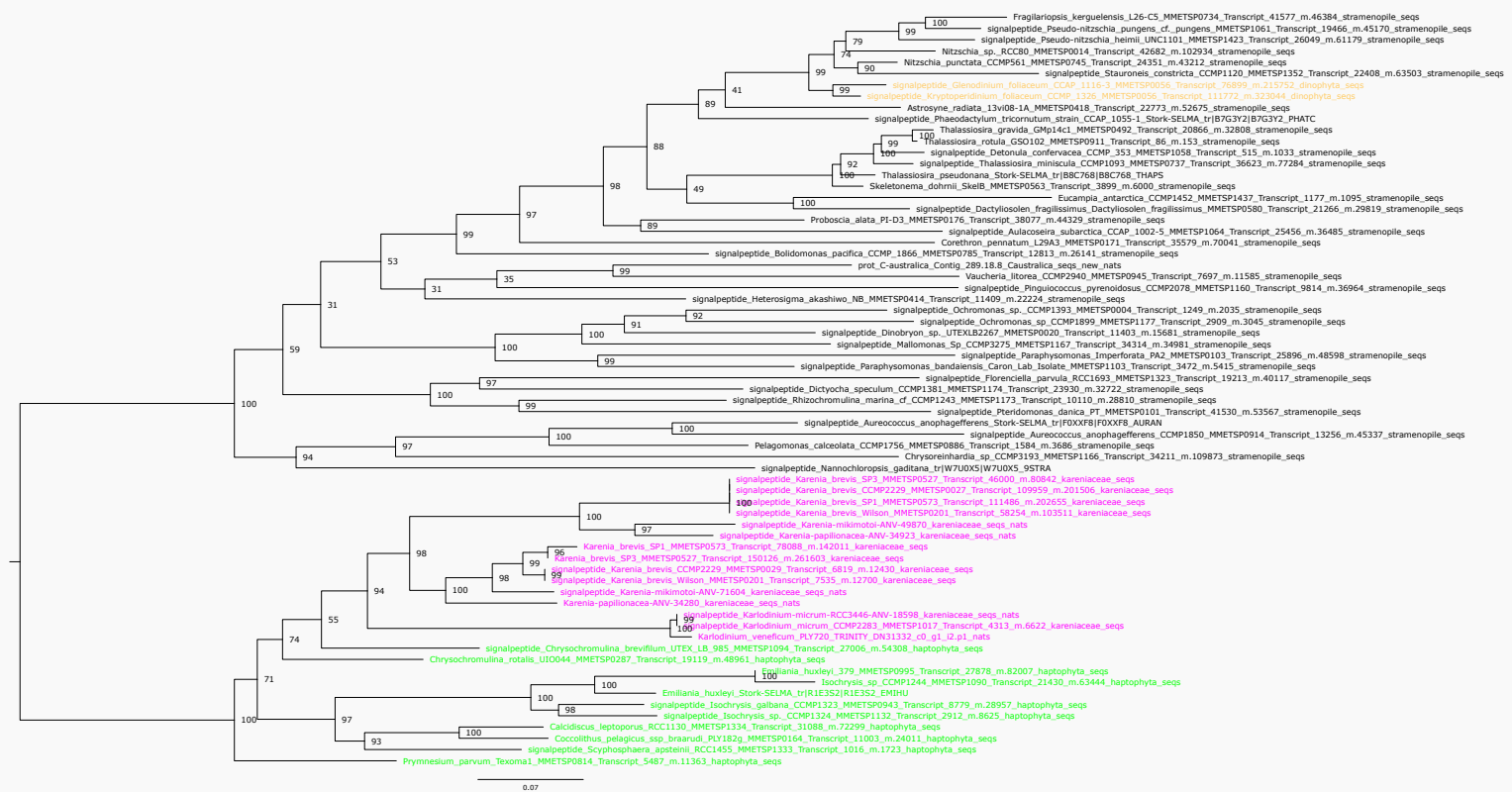



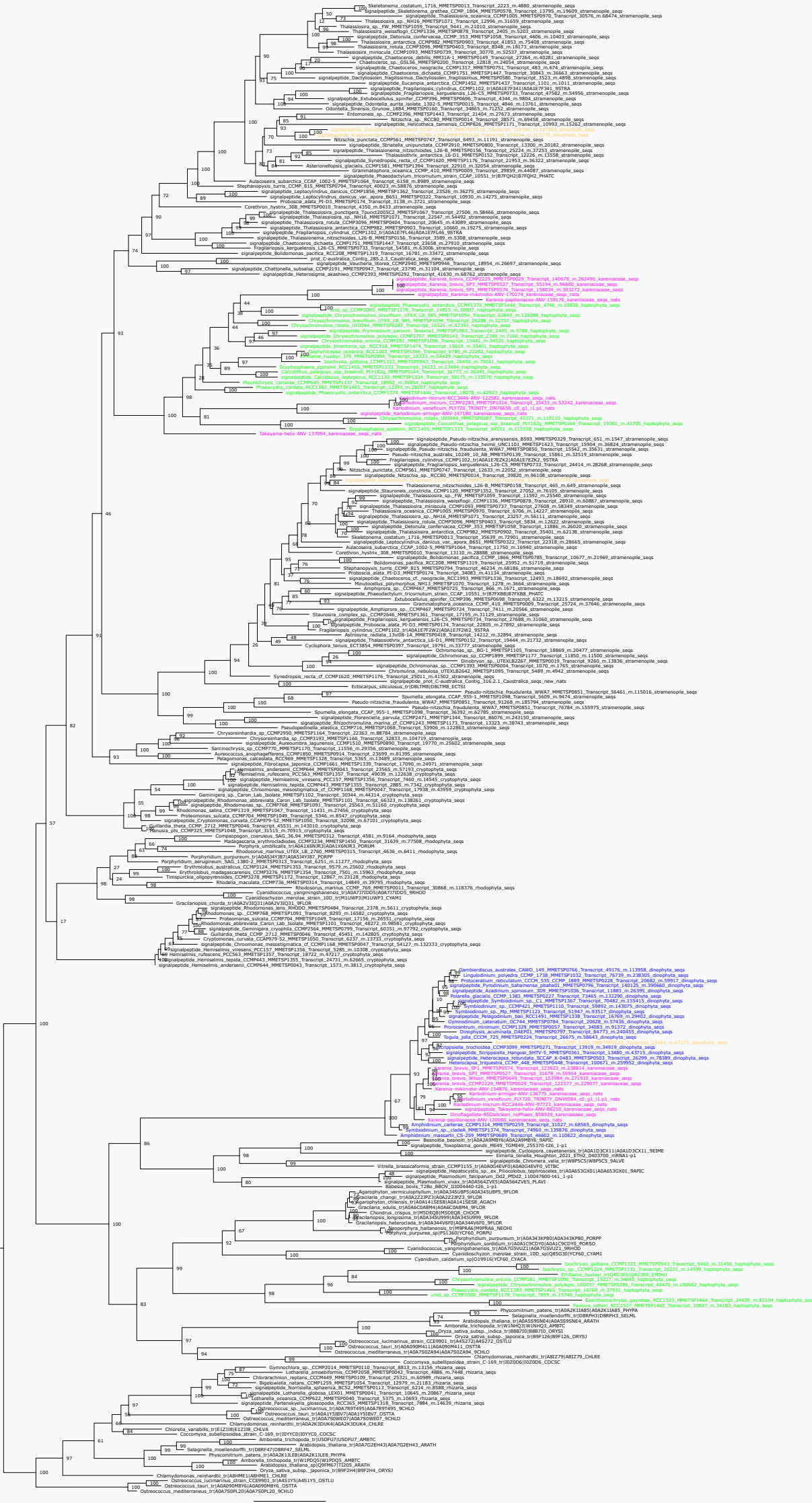

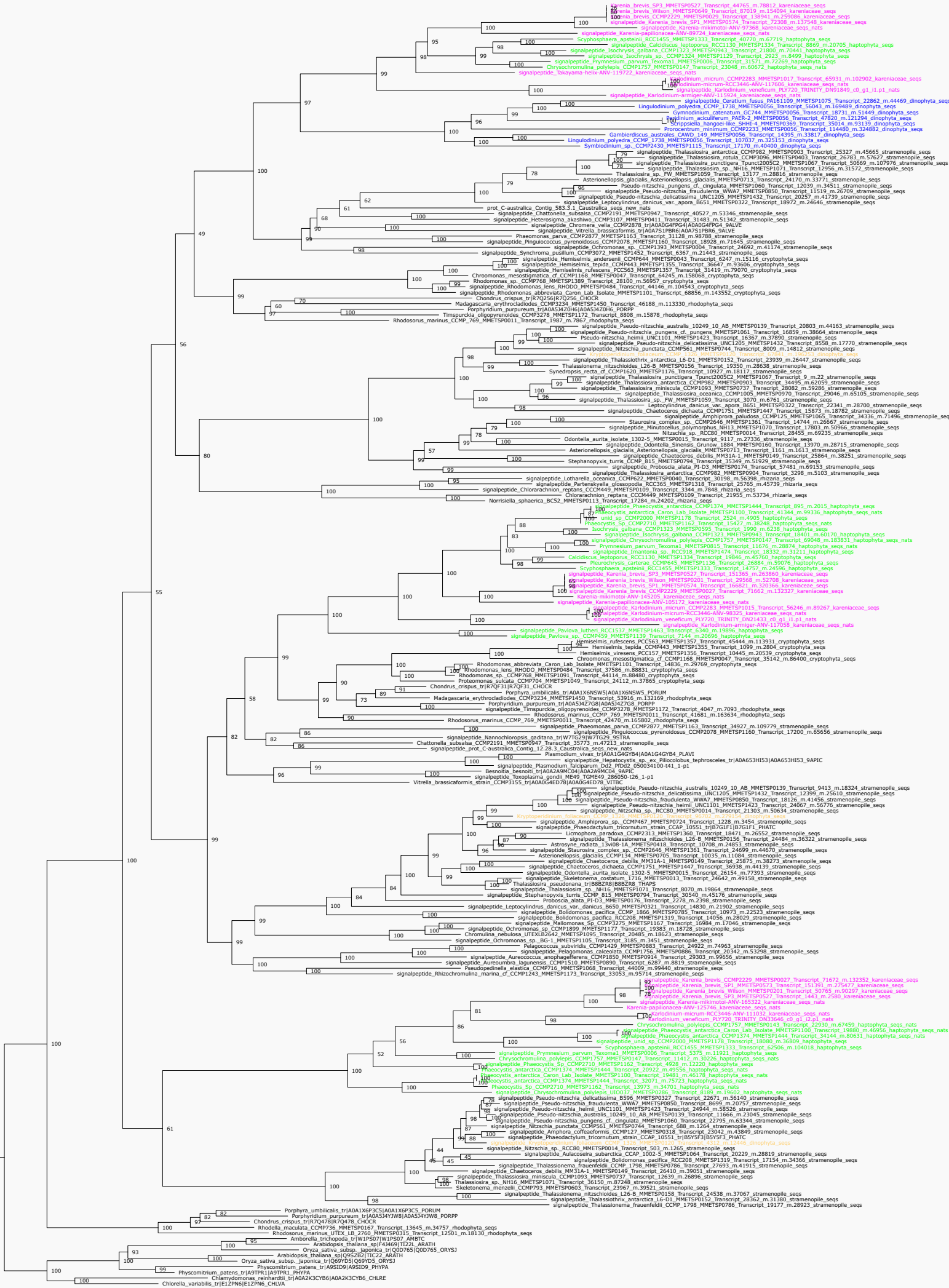

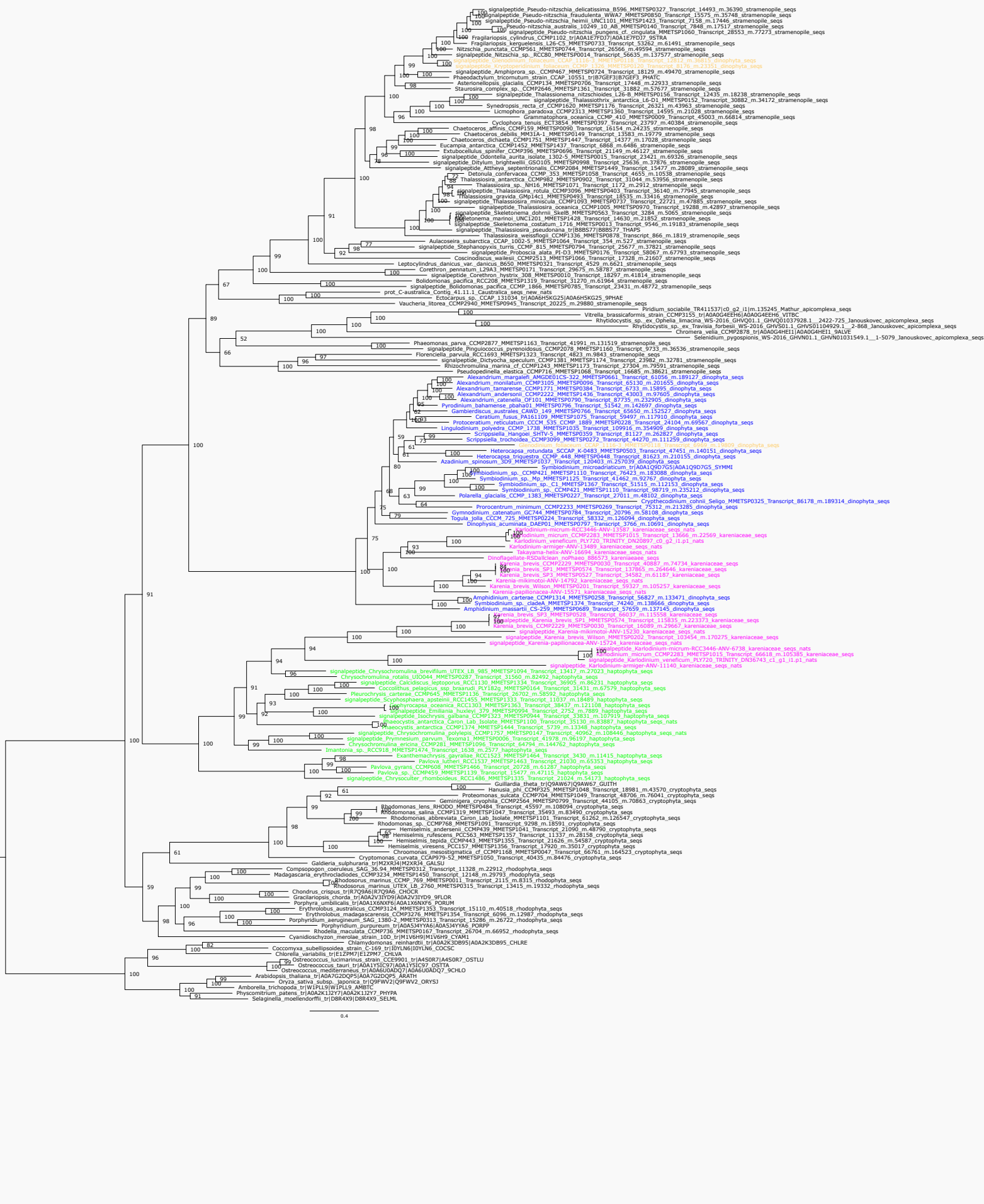



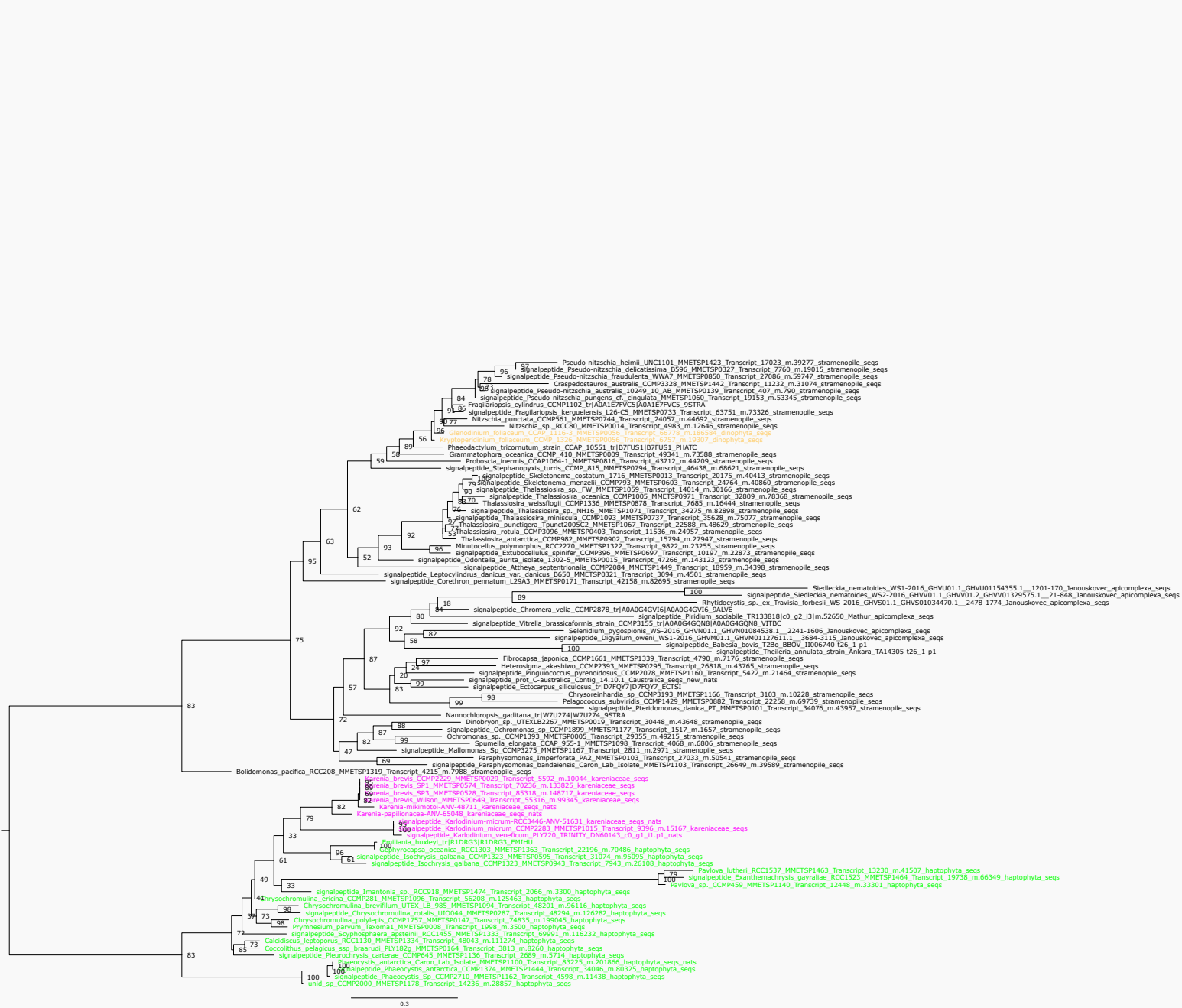

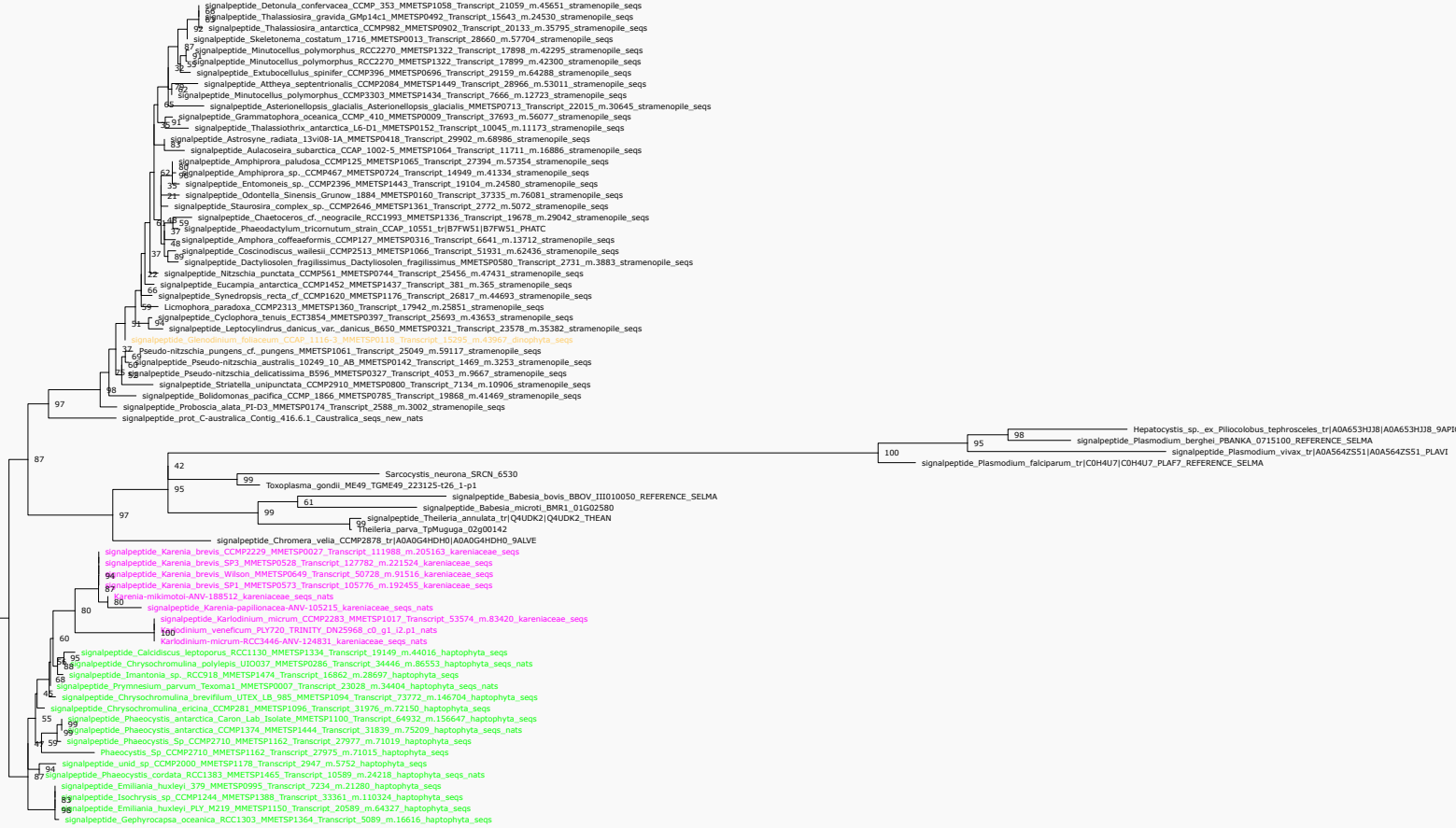

0.8

Supplement: Data S1, related to Figures 1, 2, and 3. [file EMS203403-supplement-Data_S1__related_to_Figures_1__2__and_3_.pdf]
